# Caffeine Inhibits the Activation of Hepatic Stellate Cells Induced by Acetaldehyde via Adenosine A2A Receptor Mediated by the cAMP/PKA/SRC/ERK1/2/P38 MAPK Signal Pathway

**DOI:** 10.1371/journal.pone.0092482

**Published:** 2014-03-28

**Authors:** He Wang, Wenjie Guan, Wanzhi Yang, Qi Wang, Han Zhao, Feng Yang, Xiongwen Lv, Jun Li

**Affiliations:** 1 School of Pharmacy, Anhui Medical University, Hefei, Anhui, China; 2 Institute for Liver Disease of Anhui Medical University, Hefei, Anhui, China; 3 The 105th Hospital of PLA, Hefei, Anhui, China; 4 The First Hospital of Anqing, Anqing, Anhui, China; National Institutes of Health, United States of America

## Abstract

Hepatic stellate cell (HSC) activation is an essential event during alcoholic liver fibrosis. Evidence suggests that adenosine aggravates liver fibrosis via the adenosine A2A receptor (A2AR). Caffeine, which is being widely consumed during daily life, inhibits the action of adenosine. In this study, we attempted to validate the hypothesis that caffeine influences acetaldehyde-induced HSC activation by acting on A2AR. Acetaldehyde at 50, 100, 200, and 400 μM significantly increased HSC-T6 cells proliferation, and cell proliferation reached a maximum at 48 h after exposure to 200 μM acetaldehyde. Caffeine and the A2AR antagonist ZM241385 decreased the cell viability and inhibited the expression of procollagen type I and type III in acetaldehyde-induced HSC-T6 cells. In addition, the inhibitory effect of caffeine on the expression of procollagen type I was regulated by A2AR-mediated signal pathway involving cAMP, PKA, SRC, and ERK1/2. Interestingly, caffeine’s inhibitory effect on the expression of procollagen type III may depend upon the A2AR-mediated P38 MAPK-dependent pathway. Conclusions: Caffeine significantly inhibited acetaldehyde-induced HSC-T6 cells activation by distinct A2AR mediated signal pathway via inhibition of cAMP-PKA-SRC-ERK1/2 for procollagen type I and via P38 MAPK for procollagen type III.

## Introduction

Alcoholic liver disease (ALD) encompasses a spectrum of hepatic injuries caused by long-term heavy drinking, and it is a major cause of chronic liver disease worldwide [1], [2]. In recent years, ALD has become a serious global health problem because of the striking increase in alcohol consumption [3]. Pathologic stages of ALD comprise of steatosis (alcoholic fatty liver), steato-hepatitis (alcoholic hepatitis) and liver fibrosis/cirrhosis. Steatosis and steatohepatitis represent the early stage of ALD and as precursor lesion of fibrosis/cirrhosis [4], [5]. At present, alcoholic liver fibrosis is regarded as a turning point in ALD [6]. In contrast with the traditional view that liver fibrosis/cirrhosis is an irreversible disease, recent studies have indicated that even advanced fibrosis is reversible [7], but the mechanisms are largely unknown. Therefore, the study of the pathogenesis and therapeutic targets of alcoholic liver fibrosis has received increasing attention.

The key event in the development of alcoholic liver fibrosis is the activation of hepatic stellate cell (HSC), and the activated HSCs are the major source of extracellular matrix (ECM). Although the role of HSC in the pathogenesis of hepatic fibrosis has been widely concerned, and cytokine-mediated signal transduction pathways in HSC has also been studied extensively, there is no effective therapy to reverse the development of alcohol induced hepatic fibrosis whose pathogenesis is complex and involves different molecular and biological mechanisms. It is well known that alcohol and/or its metabolites such as acetaldehyde play prominent roles in the process of alcoholic liver fibrosis [8]. Acetaldehyde, the first metabolite of ethanol, can stimulate the deposition of ECM proteins, and also stimulate type 1 collagen synthesis in cultures of rat and human HSC by increasing transcription of the specific genes [9], but the molecular mechanisms involved in the complex relationships between acetaldehyde, HSC activation and collagen production will need to be further investigated.

In recent years, the adenosine A2A receptor (A2AR) has received more attention because of its important roles in complex biological processes and a variety of fibrotic diseases [10], [11]. During ethanol metabolism, extracellular adenosine is generated by ecto-5'-nucleotidase (CD73), and adenosine production and adenosine receptor activation have been known to contribute to the development of alcohol-induced fatty liver and hepatic fibrosis [12], [13]. Chan et al. have demonstrated that adenosine and the A2AR play an active role in hepatic fibrosis by a mechanism that has been proposed to involve direct stimulation of HSC [14]. Hashmi and Sohail have also found that adenosine, acting at the A2AR in HSCs, may promote liver fibrosis progression [15], [16]. Che et al. have previously reported that the up-regulation of collagen type I mRNA and protein is A2AR-dependent, and is mediated through Gs-cAMP-PKA-SRC-ERK1/2 MAPK signaling pathways in the human hepatic cell line LX-2. However, P38 MAPK is critically involved in the A2AR-mediated regulation of collagen type III production in LX-2 cells [17].

These results mentioned above have indicated that adenosine and A2AR participate in the pathogenesis of alcoholic liver fibrosis with complex mechanisms. Taken together, these findings not only provide a better understanding of the mechanisms underlying the anti-fibrotic effects of A2AR antagonist in ALD, but also offer a satisfactory explanation for the epidemiologic finding that caffeine (1, 3, 7-trimethylxanthine), a nonselective adenosine receptor antagonist, could advantageously reduce the likelihood of ALD.

Caffeine is the most widely consumed pharmacologically active substance in the world [18]. By virtue of its purine structure, caffeine is believed to exert its pharmacological profile by blocking A2A and A1 adenosine receptors [19]. The relationship between caffeine consumption and health remains equivocal at the present time. In recent years, although caffeine has a reputation for being “bad” for health, an increasing number of epidemiological studies have reported the beneficial effects of drinking caffeine-containing beverages in the prevention of chronic liver disease, including alcoholic liver cirrhosis [20]–[22].

In 2013, the scientists first observed that caffeine stimulates the metabolization of lipids stored in liver cells and decreases the fatty liver of mice that were fed a high-fat diet. These findings indicate that moderate intake of caffeine may be beneficial in preventing and protecting against the progression of non-alcoholic fatty liver disease (NAFLD) in humans [23]. NAFLD is strongly associated with obesity, metabolic syndrome, diabetes and hyperlipidemia [24]. Although the major cause of NAFLD not due to excessive alcohol consumption, emerging evidence suggests that obesity and the metabolic syndrome exacerbate progression of ALD, and the treatment and prevention of obesity and related disorders also have a protective effect on ALD [25]. Furthermore, previous studies have shown that caffeine may improve one or more features of the metabolic syndrome in diet-induced obese rats [26]. Our previous study also reported that caffeine may represent a novel, protective strategy against alcoholic liver injury in mice by attenuating oxidative stress and inflammatory response [27]. These results further suggest that increased caffeine intake may help to reduce the risk of ALD.

In this study, HSC-T6, a rat hepatic stellate cells, was stimulated with various concentrations of acetaldehyde at different time points, which was used as an *in vitro* model for the study of alcoholic liver fibrosis. We used this cell culture model to assess whether caffeine inhibit acetaldehyde-induced activation of HSC-T6 cells via A2AR-mediated cAMP/PKA/SRC/ERK1/2/P38MAPK signaling pathway.

## Materials and Methods

### Materials and Reagents

Caffeine was purchased from Sigma Chemical Co (St. Louis, MO, USA). Acetaldehyde was purchased from Tianjin DaMao Chemical Reagent. Dimethyl sulfoxide (DMSO) was purchased from Sigma Inc. CGS21680(A2AR agonist), ZM241385(A2AR antagonist) and SB202190(P38 MAPK inhibitor) were purchased from Tocris (Ellisville, MO, USA). U0126 (MEK1/2 inhibitors), antibodies against SRC, ERK1/2, P38 MAPK, and phosphorylation-dependent antibodies against SRC, ERK1/2, P38 MAPK were purchased from Cell Signaling Technology (Beverly, MA, USA). H89 (PKA inhibitor), PP2 (SRC inhibitor) were purchased from Santa Cruz (CA, USA). Secondary antibodies including goat anti-rabbit immunoglobulin G (IgG) horse radish peroxidase (HRP) and goat anti-mouse IgG HRP were purchased from Santa Cruz Biotechnology (Santa Cruz, California, USA). RevertAidTM First Strand cDNA Synthesis Kit and Brilliant SYBR Green QPCR Master were purchased from Fermentas (CA, USA). 125I-cAMP radioimmunoassay kit was purchased from Shanghai University of Traditional Chinese Medicine (Shanghai, China). All the other chemicals used were of the highest grade available commercially.

### Cell Culture

The HSC-T6 cell line was obtained from Shanghai FuMeng Gene Biological Corporation (Shanghai, China). HSC-T6 cells were cultured in Dulbecco’s modified Eagle’s medium (DMEM, Gibco, USA), supplemented with 100 U/ml penicillin, 100 mg/ml streptomycin, 2 mM L-glutamine and 10% fetal calf serum. Cell cultures were maintained at 37°C at an atmosphere of 5% CO2.

### Cell Proliferation Assay

HSC-T6 cells were treated with acetaldehyde at different concentrations and different times to establish a cell culture model for alcohol-induced liver fibrosis in vitro. The effects of acetaldehyde on proliferation of the HSC-T6 cells were assessed by the MTT reduction assay (Sigma Chemical Co, St. Louis, MO, USA). Briefly, cells were seeded at 5×10^3^ cells in 100 μl in each well of a 96-well plate and incubated for 24 h. Six wells were used for each concentration of each compound (n  = 5 for each concentration). The cells were treated with acetaldehyde at various concentrations (25, 50, 100, 200, 400 μM) for 24 h, 48 h and 72 h. After treatment, the medium was discarded and the MTT reagent (5 mg/mL in DMEM) was added to each well and incubated for 4 h at 37°C. After incubation, the medium was discarded and 150 μl of DMSO was added to each well to dissolve the formazan crystals. The optical density (OD) was measured at 490nm and the value was compared to control cells. Experiments were repeated three times and in triplicates. Data is representated as the average percentage compared to control cells. For all subsequent experiments, 200 μM of acetaldehyde was used.

Use the same method to test the effects of caffeine, ZM241385 and CGS21680 on acetaldehyde-induced HSC-T6 cells. The cells were treated with caffeine at various concentrations (0.5, 1, 2, 4, 8 mM), ZM241385 at various concentrations (1, 10, 100, 1000, 10000 nM) and CGS21680 at various concentrations (1, 10, 100, 1000, 10000 nM) after 1 h of treatment with 200 μM acetaldehyde for 24 h, 48 h, 72 h. The OD was measured at 490nm. The percentage of cell viability was calculated according to the following formula: cell viability %  =  (T/C) ×100%, where T and C refer to the mean OD of experiment groups and cell control, respectively. Experiments were also repeated three times and in triplicate. Data is represented as the average percentage compared to control cells. For all subsequent experiments, 4 mM of caffeine was used.

### Quantitative RT-PCR for A2AR, Procollagen Type I, Procollagen Type III and PKA Gene Expression

Total RNA was extracted from HSC-T6 cells using TRIzol reagent (Invitrogen Co. USA). The first-strand cDNA was synthesized from total RNA using AMV Reverse Transcriptase (Fermentas) according to the manufacturer's protocol. Quantitative real-time PCR analyses for mRNA of A2AR, procollagen type I, procollagen type III, PKA and β-actin were performed by using ABI RT-PCR kits (ABI, USA). The mRNA level of β-actin was used as an internal control. qRT-PCR was performed under standard protocol using the following primers:

β-actin(forward:5'-ACCACAGCTGAGAGGGAAATCG-3'; reverse:5'-AGAGGTCTTTACGGATGTCAACG-3'),

A2AR(forward:5'-CCATGCTGGGCTGGAACA-3'; reverse:5'-CGTTCGTGTTACTGCCCCTTC-3'),

procollagen type I (forward:5'-GATCCTGCCGATGTCGTCAT-3';

reverse:5'-TGTAGGCTACGCTGTTCTTGCA-3'),

procollagen type III (forward:5'-ATGGTGGCTTTCAGTTCAGC-3';

reverse: 5'-TGGGGTTTCAGAGAGTTTGG-3'),

PKA (forward: 5'-GCTGG CTTTGATTTACGG-3';

reverse:5'-GATGTTTCGCTTGAGGATA-3'). qRT-PCR was performed at 94°C for 5 min, followed by 40 cycles of amplification at 94°C for 40s, 51°C for 40s and 72°C for 1 min by using ABI9700. The results were performed in triplicate and repeated at least three times.

### cAMP Assay

HSC-T6 cells were grown in 12-well plates (5×104 cells/well). Culture-treated HSC-T6 cells were lysed and assayed in triplicate. The cAMP concentrations were measured using a cAMP radio immunoassay kit according to the manufacturer’s instructions.

### Western Blot Analysis

Western blotting was conducted according to standard protocols. Culture-treated HSC-T6 cells were washed twice with PBS, and total cell protein extracts were prepared and lysed in ice-cold RIPA buffer for 15 min on ice. The lysates were sonicated for 2 s, to shear the DNA to reduce its viscosity, followed by a centrifugation step at 13,000 ×g for 30 min at 4°C. Protein concentrations were determined using a BCA protein assay kit (Boster, China). Cell lysates were mixed with 5 × Laemmli buffer and heated for 10 min at 99°C. Samples were separated by sodium dodecylsulfate polyacrylamide gel electrophoresis and then blotted protein onto a PVDF membrane (Millipore Corp, Billerica, MA, USA). After blocking, PVDF blots were incubated for 1 h with a primary antibody diluted in TBS/Tween20 (0.075%) containing 3% Marvel. Anti-phospho-SRC, anti-SRC, anti-phospho-ERK, anti-ERK, anti-phospho-P38 and anti-P38 were diluted to 1∶500. Horseradish peroxidase conjugated anti-mouse and anti-rabbit antibodies were used as secondary antibodies correspondingly. After washing with TBS/Tween-20 for four times, the membranes were developed with distilled water for detection of antigen using the enhanced chemiluminescence system. Proteins were visualized with ECL-chemiluminescent kit (ECL-plus, Thermo Scientific). Experiments were repeated at least three times from three independent protein extracts.

### Statistical Analysis

All results are expressed as the mean ± SE. Statistical significance was identified by either a Student’ t-test for comparison between means or one-way analysis of variance with a post hoc Dunnett’s test. P<0.05 was considered statistically significant.

## Results

### *In Vitro* Cell Culture Model for Alcohol Induced Liver Fibrosis

**Figure 1:**
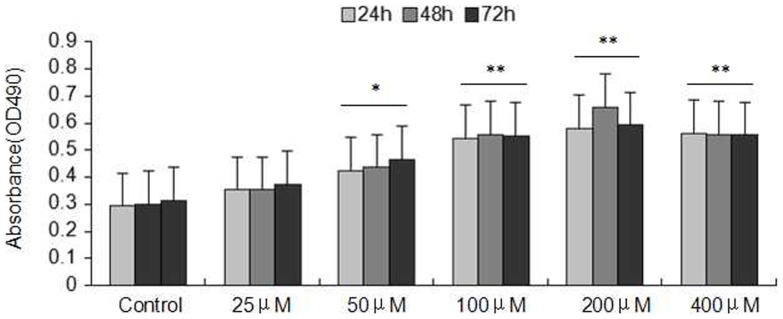
Effect of acetaldehyde (25, 50, 100, 200, 400 μM ) on cell proliferation in HSC-T6 cells for different time(24 h, 48 h, 72 h). Acetaldehyde significantly increased proliferation of HSC-T6 cells. The role of acetaldehyde in regulating HSC-T6 cells proliferation was tested by MTT assay. The dates represent the mean ± SD of three different experiments. ^*^ indicate P<0.05, ^**^ indicate P<0.01 versus control group.

Acetaldehyde, the major product of alcohol metabolites, can directly and (or) indirectly promote HSC activation. To mimic the model of alcohol-induced liver fibrosis, HSC-T6 cells were treated with different concentrations of acetaldehyde. Cell proliferation was determined by the MTT method. Cell proliferation of HSC-T6 cells treated with acetaldehyde at concentration of 25, 50, 100, 200, and 400 μM for 24 h, 48 h and 72 h, respectively, were investigated. As shown in Figure 1, acetaldehyde at 50, 100, 200, and 400 μM significantly increased HSC-T6 cells proliferation, and a maximum increase in proliferation was observed at 48 h after exposure to 200 μM acetaldehyde. Then we chose 200 μM acetaldehyde to intervene the cells in the further experiments.

### Caffeine and A2AR Modulators Regulated Acetaldehyde-Induced HSC-T6 Cells Proliferation

**Figure 2:**
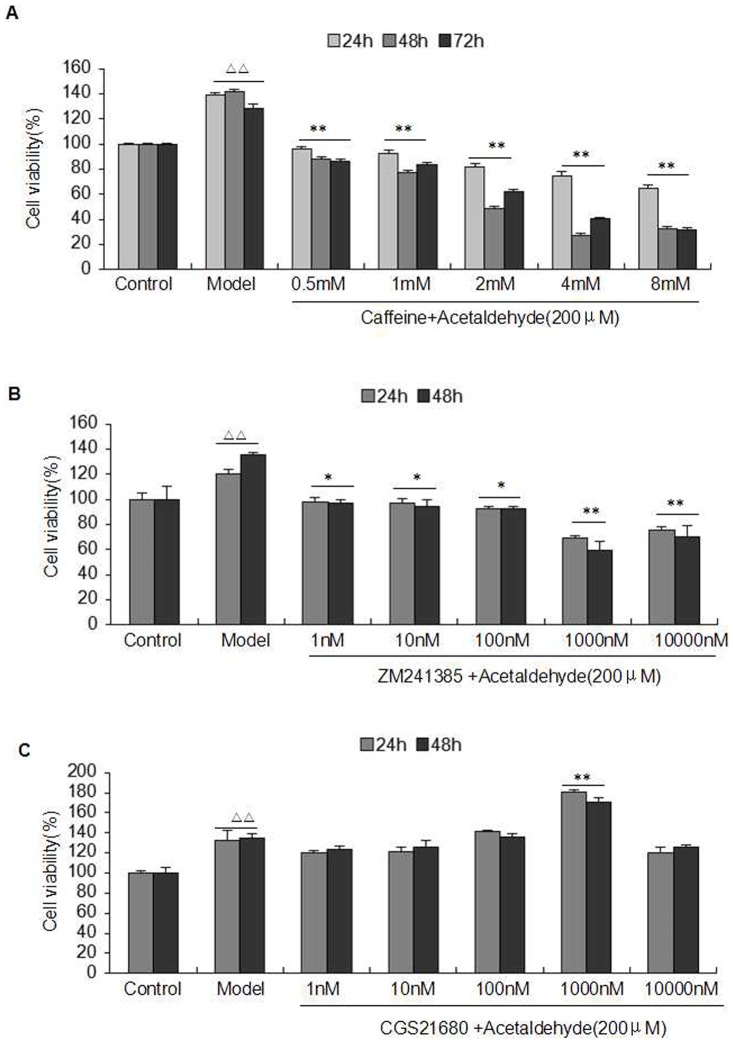
Effect of caffeine and A2AR modulators on the acetaldehyde-induced HSC-T6 cells proliferation. (**A**) HSC-T6 cells were treated with caffeine (final concentration of caffeine was 0.5, 1, 2, 4, 8 mM, respectively) at the indicated times (24h, 48h, 72h). (**B**) HSC-T6 cells were treated with ZM24138 (final concentration of ZM24138 was 1, 10, 100, 1000, 10000 nM, respectively) at the indicated times (24h, 48h). (**C**) HSC-T6 cells were treated with CGS21680 (final concentration of CGS21680 was 1, 10, 100, 1000, 10000 nM, respectively) at the indicated times (24h, 48h). Cell viability was determined by MTT assay. Each column represents the mean±S.D. of six separate experiments. ^△△^ indicates P<0.01 versus control group. ^*^ indicates P<0.05, ^**^ indicates P<0.01 versus model group (acetaldehyde 200 μM only).

Acetaldehyde-induced HSC-T6 cells were cultured for 24 h, 48 h, and 72 h with caffeine at various concentrations ranging from 0.5 mM to 8 mM. Our data showed that the cell viability was significantly decreased in the caffeine-treated group as compared with that of model group (acetaldehyde only). The maximum inhibitory effects were achieved after 48h of treatment with 4 mM caffeine (Figure 2A). As shown in Figure 2B and Figure 2C, the cell viability in the presence of ZM241385 (1 μM) decreased the most at 48 h, and CGS21680 (1 μM) markedly promoted the cell viability of HSC-T6 at 24 and 48 h, respectively. The results showed that the proliferation of HSC-T6 cells was inhibited by caffeine and ZM241385, while CGS21680 could further promote HSC-T6 cell proliferation.

### Effects of Caffeine and A2AR Modulators on Procollagen Type I and Procollagen Type III mRNA Expression

**Figure 3:**
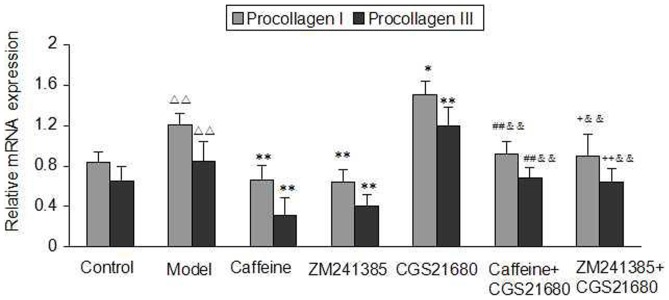
Effect of caffeine and A2AR modulators on expression of procollagen type I and procollagen type III mRNA in acetaldehyde-induced HSC-T6 cells. HSC-T6 cells were treated with 200 μM acetaldehyde, 4 mM caffeine, 1 μM CGS21680, 1 μM ZM241385, 1 μM CGS21680 plus 4 mM caffeine or 1 μM CGS21680 plus 1 μM ZM241385 for 48h before harvesting and measurement of collagen production in the cells as described. Data are expressed as the mean ±S.D. (n = 3). ^△△^ indicates P<0.01 versus control group. ^*^ indicates P<0.05, ^**^ indicates P<0.01 versus model group (acetaldehyde 200 μM only). ^##^ indicates P<0.01 versus caffeine group. ^+^ indicates P<0.05, ^++^ indicates P<0.01 versus ZM241385 group. ^&&^ indicates P<0.01 versus CGS21680 group.

We then evaluated the hypothesis that caffeine and the A2AR antagonist can inhibit collagen synthesis of acetaldehyde-induced HSC-T6 cells. In response to liver injury, HSC-T6 cells undergo an activation process in which they proliferate and synthesize a fibrotic matrix rich in procollagen type I and type III [17]. Representative examples of procollagen I and procollagen III mRNA expression in different groups are shown in Figure 3. The expression of procollagen I and III mRNA of HSC-T6 cells stimulated by acetaldehyde was decreased markedly while simultaneously being exposed to either caffeine or ZM241385. In contrast, CGS21680 stimulated the expression of procollagen I and III mRNA production as compared with the model group. However, the increase in HSC-T6 cells collagen production was almost completely abrogated by caffeine and by ZM241385. These results indicated that the mechanism of caffeine-inhibited collagen production may be involved in modulating adenosine A2AR-mediated signal pathway.

### Quantification of the Expression of the A2AR in Acetaldehyde-Induced HSC-T6 Cells

**Figure 4:**
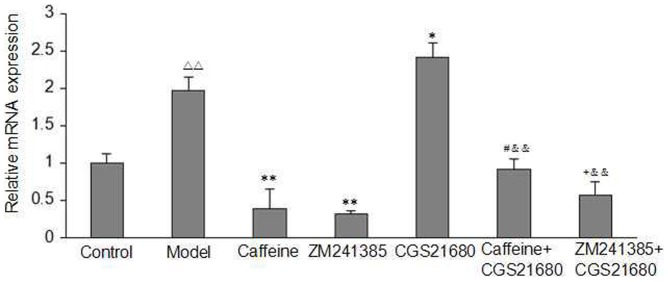
Effect of caffeine and A2AR modulators on A2AR mRNA Expression in acetaldehyde-induced HSC-T6 cells. Expression of A2AR mRNA was analysed by qRT-PCR. HSC-T6 cells were treated with 200 μM acetaldehyde, 4 mM caffeine, 1 μM CGS21680, 1 μM ZM241385, 1 μM CGS21680 plus 4 mM caffeine or 1 μM CGS21680 plus 1 μM ZM241385 for 48h before harvesting and measurement of A2A Receptors in the cells as described. The data are presented as mean ±S.D. of triplicate cultures in four separate experiments. ^△△^ indicates P<0.01 versus control group. ^*^ indicates P<0.05, ^**^ indicates P<0.01 versus model group (acetaldehyde 200 μM only). ^#^ indicates P<0.05 versus caffeine group. ^+^ indicates P<0.05 versus ZM241385 group. ^&&^ indicates P<0.01 versus CGS21680 group.

To investigate whether A2AR expression is altered in acetaldehyde-induced HSC-T6 cells, we utilized qRT-PCR to quantify the levels of A2AR mRNA transcripts. As shown in Figure 4, acetaldehyde (200 μM) increased the A2AR mRNA expression in HSC-T6 cells. Both caffeine and ZM241385 treatment inhibited the mRNA expression of A2A as compared with the model group, whereas CGS21680 had the opposite effect. Likewise, caffeine or ZM241385 abolished the effect of CGS21680 on A2AR mRNA expression.

### Caffeine Inhibited Acetaldehyde-Induced HSC-T6 Cells Proliferation via the cAMP/PKA/SRC/ERK1/2/P38 MAPK Pathway

**Figure 5:**
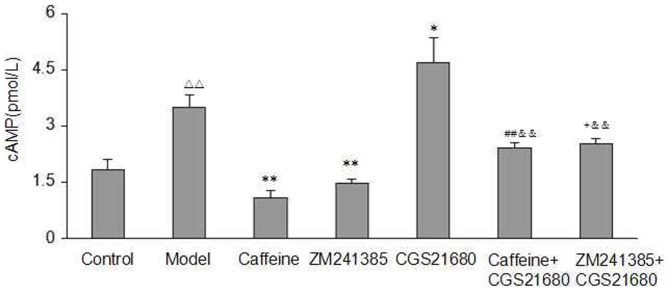
Effect of caffeine and A2AR modulators on the concentration of cAMP in acetaldehyde-induced HSC-T6 cells. HSC-T6 cells were treated with 200 μM acetaldehyde, 4 mM caffeine, 1 μM CGS21680, 1 μM ZM241385, 1 μM CGS21680 plus 4 mM caffeine or 1 μM CGS21680 plus 1 μM ZM241385 for 48h before the cAMP concentration was tested. ^△△^ indicates P<0.01 versus control group. ^*^ indicates P<0.05, ^**^ indicates P<0.01 versus model group (acetaldehyde 200 μM only). ^##^ indicates P<0.01 versus caffeine group.^ +^ indicates P<0.05 versus ZM241385 group. ^&&^ indicates P<0.01 versus CGS21680 group.

**Figure 6:**
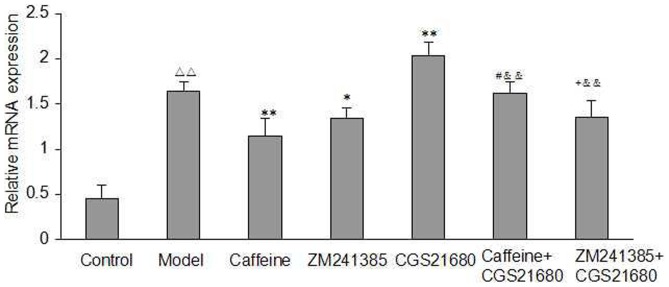
Effect of caffeine and A2AR modulators on the mRNA Expression of PKA in acetaldehyde-induced HSC-T6 cells. HSC-T6 cells were treated with 200 μM acetaldehyde, 4 mM caffeine, 1 μM CGS21680, 1 μM ZM241385, 1 μM CGS21680 plus 4 mM caffeine or 1 μM CGS21680 plus 1 μM ZM241385 for 48h before harvesting and measurement of PKA in the cells as described. Data are expressed as the mean ±S.D. (n = 3). ^△△^ indicates P<0.01 versus control group. ^*^ indicates P<0.05, ^**^ indicates P<0.01 versus model group (acetaldehyde 200 μM only). ^#^ indicates P<0.05 versus caffeine group. ^+^ indicates P<0.05 versus ZM241385 group. ^&&^ indicates P<0.01 versus CGS21680 group.

The signaling pathways activated by A2AR are mediated via Gs protein-dependent adenylate cyclase (AC) activation, which lead to the increase of intracellular cAMP concentration, and thereby activation of PKA. Here we examined the influence of caffeine and the A2AR agonist and antagonist on the level of cAMP/PKA signaling in acetaldehyde-induced HSC-T6 cells. As shown in Figure 5 and Figure 6, acetaldehyde increased intracellular cAMP concentration and PKA activity as compared with the control group, and that this increase could be inhibited potently by caffeine and ZM241385. As expected, the A2AR agonist CGS21680 led to a significant increase in the level of cAMP/PKA signaling as compared with the model group, which was abolished by the addition of caffeine or ZM241385 to the cells.

**Figure 7:**
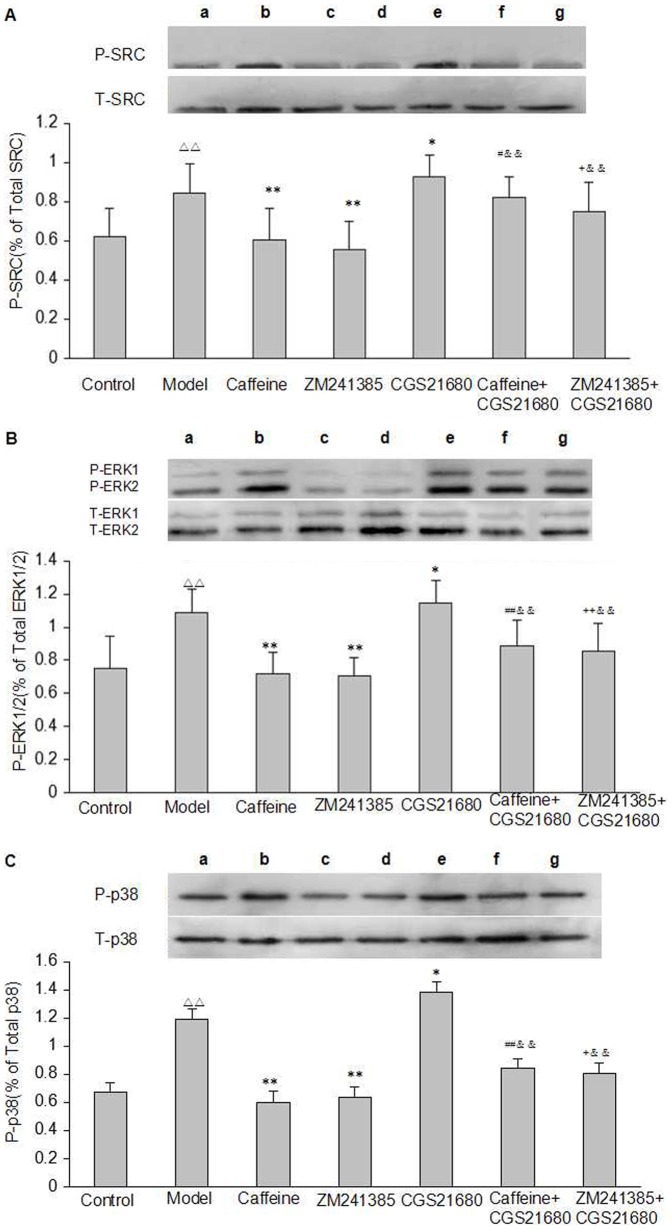
Effect of caffeine and A2AR modulators on the phosphorylation of SRC, ERK1/2, and P38 MAPK pathway in acetaldehyde-induced HSC-T6 cells. (A) SRC Kinase. (B) ERK1/2 MAPK. (C) P38 MAPK. HSC-T6 cells were treated with 200 μM acetaldehyde, 4 mM caffeine, 1 μM CGS21680, 1 μM ZM241385, 1 μM CGS21680 plus 4 mM caffeine or 1 μM CGS21680 plus 1 μM ZM241385 for 48h before harvesting and measurement of proteins expression in cells as described. Data are expressed as the mean ±S.D. (n = 3). ^△△^ indicates P<0.01 versus control group. ^*^ indicates P<0.05, ^**^indicates P<0.01 versus model group (acetaldehyde 200 μM only). ^#^ indicates P<0.05, ^##^ indicates P<0.01 versus caffeine group. ^+^ indicates P<0.05, ^++^ indicates P<0.01 versus ZM241385 group. ^&&^ indicates P<0.01 versus CGS21680 group. a: Control, b: Model, c: Caffeine, d: ZM241385, e: CGS21680, f: Caffeine+CGS21680, g: ZM241385+CGS21680.

Activation of cAMP/PKA pathway which, in turn, phosphorylate downstream signaling molecules. As shown in Figure 7, after exposure to acetaldehyde, the phosphorylation of SRC, ERK1/2, and P38 MAPK was increased compared to control group, and the magnitude of the increase was greater in CGS21680-treated cells than in model group. However, these effects were prevented by caffeine and by ZM241385. These data further demonstrated that caffeine may be via A2AR mediated signaling pathway to inhibit the phosphorylation of SRC, ERK1/2, and P38 MAPK in acetaldehyde-induced HSC-T6 cells.

### Involvement of Protein Kinases in Caffeine Decreased Acetaldehyde-Induced Production of Procollagen Type I and Procollagen Type III

**Figure 8:**
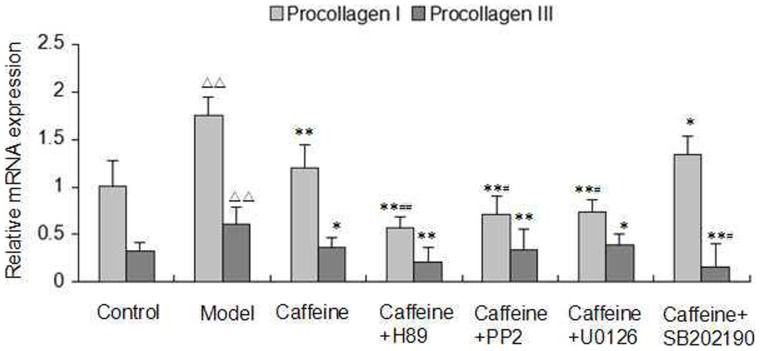
Effect of inhibitors of PKA, SRC, ERK1/2, and P38 MAPK on caffeine-inhibited procollagen type I and procollagen type III mRNA expression in acetaldehyde-induced HSC-T6 cells. HSC-T6 cells were treated with 4 mM caffeine alone or in combination with 20 μM H89, 20 μM PP2, 20 μM U0126, and 20 μM SB202190 respectively for 48 h. Data are expressed as the mean ±S.D. (n = 3). ^△△^ indicates P<0.01 versus control group. ^*^ indicates P<0.05, ^**^ indicates P<0.01 versus model group (acetaldehyde 200 μM only). ^#^ indicates P<0.05, ^##^ indicates P<0.01 versus caffeine group.

To better understand the role of A2AR-mediated signal transduction in the process of caffeine down-regulates collagen production, we used a series of kinase inhibitors, which led to the disruption of the protein phosphorylation pathway in acetaldehyde-induced HSC-T6 cells. We were interested to find that combined treatment with caffeine and PKA inhibitor H89, SRC inhibitor PP2, or ERK1/2 inhibitor U0126, respectively, had a strong inhibition of procollagen I expression than caffeine alone, while P38 MAPK inhibitor SB202190 did not potentiate this inhibitory effects. By contrast, combined treatment with SB202190 further augmented the inhibitory effect of caffeine on the expression of procollagen III (Figure 8). These data demonstrated that caffeine may inhibit the production of both procollagen I and III by different signaling pathways.

### Caffeine Inhibited Both Procollagen Type I and Type III Expression by Distinct Pathways

**Figure 9:**
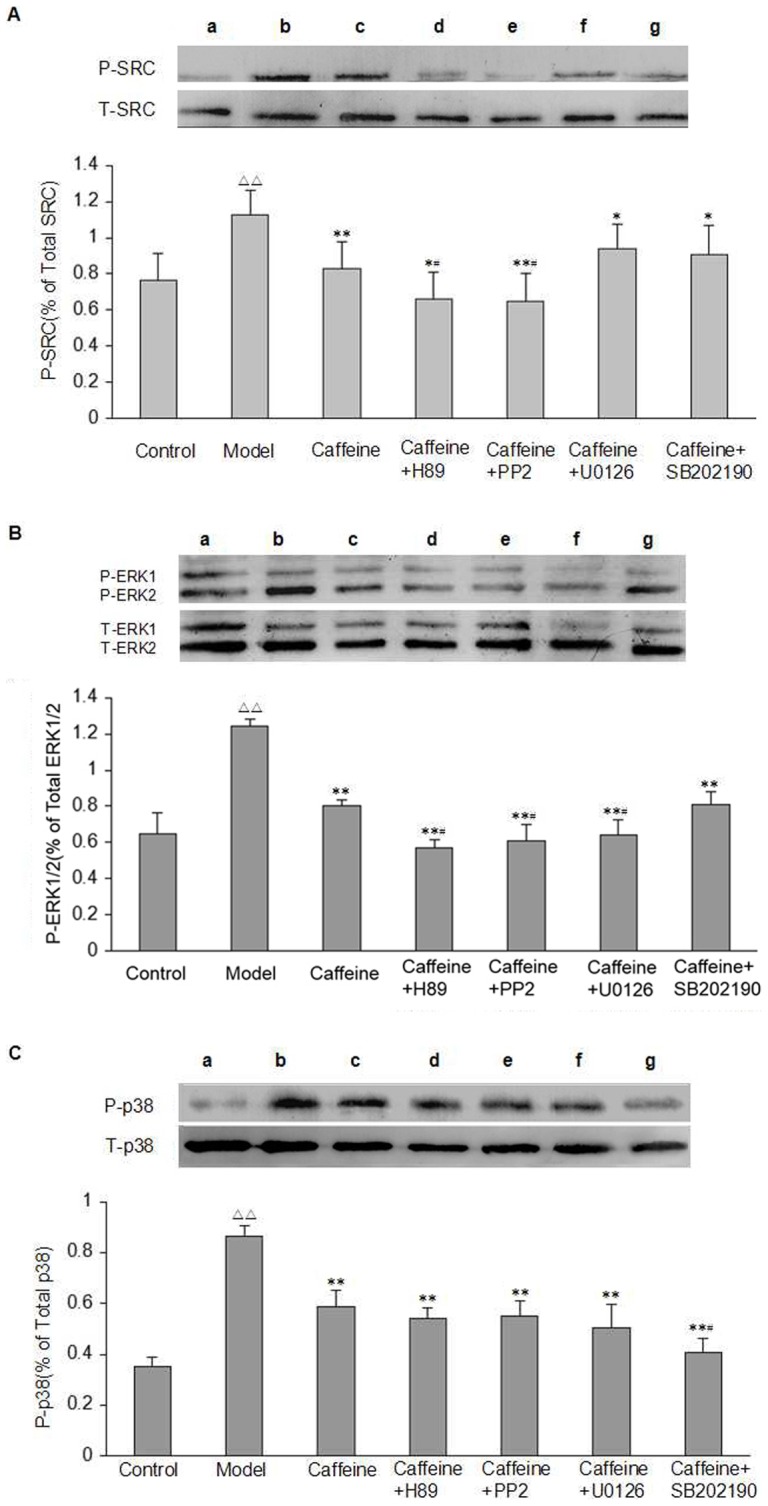
Effect of H89, PP2, U0126, and SB202190 on the phosphorylation of SRC, ERK1/2, and P38 MAPK in acetaldehyde-induced HSC-T6 cells treated with caffeine. HSC-T6 cells were treated with 4 mM caffeine alone or in combination with 20 μM H89, 20 μM PP2, 20 μM U0126, and 20 μM SB202190 respectively for 48 h. Data are expressed as the mean ±S.D. (n  = 3). (A) SRC Kinase. (B) ERK1/2 MAPK. (C) P38 MAPK. ^△△^ indicates P<0.01 versus control group. ^*^ indicates P<0.05, ^**^ indicates P<0.01 versus model group (acetaldehyde 200 μM only). ^#^ indicates P<0.05, ^##^indicates P<0.01 versus caffeine group. a: Control, b: Model, c: Caffeine, d: Caffeine+H89, e: Caffeine+PP2, f: Caffeine+U0126, g: Caffeine+ SB202190.

As described in Figure 7A, 7B and 7C, caffeine inhibited acetaldehyde-stimulated phosphorylation of SRC, ERK1/2, and P38 MAPK. Here, we found that the addition of inhibitors of PKA and SRC to the caffeine-treated cells further decreased the level of phosphorylated SRC, while inhibitors of ERK1/2 did not affect the phosphorylation of SRC (Figure 9A). Furthermore, combined use of caffeine and inhibitors of PKA, SRC, and ERK1/2 down-regulated the phosphorylation of ERK1/2 as compared with the caffeine group (Figure 9B). In contrast, the simultaneous treatment with caffeine and SB202190 did not interfere with any of these signaling events. In addition, we also found that combined caffeine with SB202190 reduced P38 MAPK phosphorylation as compared with the caffeine group, but the addition of inhibitors of PKA, SRC, and ERK1/2 to caffeine did not affect caffeine’s inhibitory effect on the phosphorylation of P38 MAPK (Figure 9C). These findings parallelled the inhibitory effects of caffeine on procollagen I and III production in HSC-T6 cells, and demonstrated that caffeine inhibited the production of procollagen I via PKA-SRC-ERK1/2 and procollagen III via P38 MAPK signaling pathway.

## Discussion

Alcohol abuse and alcohol dependence affect millions of individuals worldwide. Acute and chronic exposure to ethanol initiates and promotes the development of ALD from simple steatosis to fibrosis and cirrhosis [28]. A key event in the pathogenesis of hepatic fibrosis is the transition of quiescent HSCs into activated myofibroblasts, with an increase in the expression of collagen production, which results in hepatic fibrosis [29].

Acetaldehyde, the primary oxidative metabolite of alcohol, induces the activation of HSC, leading to up-regulation of collagen synthesis and ultimately causing hepatic fibrosis [30]–[32]. Ethanol induces liver fibrosis by several means that include, among others, the direct fibrogenic action of acetaldehyde on HSC. However the intracellular signaling pathways involved in mediating these effects are not well understood. In this study, in vitro HSC-T6 cells culture model was established following exposure to acetaldehyde, which would better mimic HSC activation seen in vivo model of alcoholic liver fibrosis. It was important to note that acetaldehyde could significantly increase the HSC-T6 cells proliferation, and cell proliferation reached a maximum at 48 h after exposure to 200 μM acetaldehyde, consistent with the majority of previous studies [33], [34].

In recent years, more and more studies have reported that coffee consumption reduces the risk of liver diseases [22]. The active ingredient in coffee is caffeine, which is the most commonly consumed psychoactive substance in the world. Furthermore, caffeine is a non-selective adenosine receptor antagonist, which binds with very similar (relatively high) affinity to both adenosine A1 and A2A receptors. The most likely mechanism of action of caffeine at physiological concentrations is the antagonism of adenosine receptors [35]. Recent experiments have shown that treatment with A2AR antagonist both prevented and reversed the ability of ethanol to exacerbate liver fibrosis [36]. Results from this report provided a novel insight into mechanisms by which the presence of caffeine may decrease the risk of chronic liver diseases by acting as an A2AR antagonist. Therefore, based on those previous results, we treated HSC-T6 cells with various concentrations of acetaldehyde, caffeine, ZM241385 and CGS21680 at different time points. Our results showed that the viability of HSC-T6 cells stimulated by acetaldehyde was decreased markedly while simultaneously being exposed to either caffeine or A2AR antagonist ZM241385. In contrast, CGS21680, a selective A2AR agonist, could further promote the proliferation of HSC-T6 induced by 200 μM acetaldehyde. The results demonstrated that caffeine had inhibitory effect on the proliferation of HSC-T6 cells induced by acetaldehyde.

Alcoholic liver fibrosis is characterized by excessive deposition of ECM, especially collagen types I and III [37]. HSC activation is the initial event that triggers the process of fibrogenesis, and activated HSC is the primary fibrogenic cell responsible for much of the collagen synthesis [38]. Numerous studies have shown that both ethanol and acetaldehyde induce the expression of type I and III collagen in HSCs [39]. These findings are consistent with our results that acetaldehyde and A2AR agonist CGS21680 in vitro increased mRNA expression of procollagen I and III. In the meantime, we observed that caffeine and A2AR antagonist ZM24138 also significantly inhibited the expression of procollagen I and III. The mechanism may be involved in modulating A2AR-mediated signal pathway.

Adenosine is a regulatory nucleoside that is generated in response to cellular stress and damage and is therefore increased during episodes of tissue hypoxia and inflammation [40]. Adenosine concentrations in the liver are increased in animal models of alcoholic liver injury and ethanol is well known to stimulate increased extracellular adenosine concentration *in vitro* through its action on the nucleoside transporter [14]. Extracellular adenosine signals through four G-protein coupled adenosine receptors, A1, A2A, A2B, and A3 [41]. Recent evidence has accumulated to suggest an important regulatory role of A2AR in the pathogenesis of alcohol-induced liver fibrosis [14]. Data from several laboratories have shown that adenosine stimulates HSC-mediated fibrosis of the liver [42]. Although the modulation of these effects by adenosine appears to occur predominantly via A2ARs, the results have not been carefully validated and the participant signaling pathways in HSC have not been unequivocally established. As expected, our quantitative RT-PCR results indicated that the mRNA for A2AR was present in HSC-T6 cells, and a much stronger increase in A2AR mRNA levels was observed in acetaldehyde-activated HSC-T6 cells than in control cells, whereas this effect was abolished by caffeine and ZM241385. A2ARs are Gs-coupled receptors whose activation stimulate the formation of intracellular cAMP [43], and the additional downstream pathways include PKA, CREB, and P38 activation [44]. Our data indicated that A2AR may be functionally present in HSC-T6 cells, thus adenosine accumulation induced by acetaldehyde applied to HSC-T6 could bind A2AR that couple to Gs, simultaneously activating multiple downstream signaling pathways. Our data further demonstrated that treatment of HSC-T6 with acetaldehyde and the A2AR agonist CGS21680 led to a significant increase in intracellular cAMP concentration and PKA activity, which was blocked by caffeine and ZM241385, indicating functional coupling of the A2AR to the Gs-AC signaling pathway in the HSC-T6 cells.

Previous studies have assessed that a number of downstream signals play a role in the stimulation of collagen production by both primary HSCs and LX-2 cells including activation of the downstream targets of ERK1/2 and P38 MAPK [17]. It has also been reported that activation of A2AR increases the activity of ERK1/2 [45], [46], at least in part, through the Src/ERK1/2 signaling pathway [47]. It is now clear that P38 MAPK is also associated with the promotion of liver fibrosis [48], [49], and previous reports have indicated that the MAPK and cAMP/PKA pathways exhibit extensive cross-talk [50]. However, it is not clear if A2AR-mediated activation of ERK1/2 and MAPK is required for acetaldehyde-induced HSC proliferation. We provided data in this work that stimulation of HSC-T6 with acetaldehyde (200 μM) significantly increased SRC, ERK1/2, and P38 MAPK phosphorylation, and the magnitude of the increase was greater in CGS21680-treated cells than in model group. However, the apparent decrease could be detected following treatment with caffeine and ZM241385. Thus, it is possible that caffeine may be via A2AR mediated signaling pathway to inhibit the phosphorylation of SRC, ERK1/2, and P38 MAPK in acetaldehyde-induced HSC-T6 cells.

To elucidate the mechanism of the observed relationship between the suppressive effects of caffeine and A2AR-mediated signaling pathway downstream, a series of kinase inhibitors were applied to the present study. As previously described, caffeine significantly inhibited acetaldehyde-induced procollagen I and III mRNA expression. Interestingly, the use of caffeine combined with PKA inhibitor H89, SRC inhibitor PP2, and ERK1/2 inhibitor U0126 for the treatment of the cells showed more significant inhibition of procollagen I expression than caffeine alone, but P38 MAPK inhibitor SB202190 did not potentiate the inhibitory effects. In contrast, only the combined caffeine and SB202190 treatment further augmented the inhibitory effect of caffeine on the mRNA expression of procollagen type III. These results indicated that in acetaldehyde-induced HSC-T6 cells, caffeine could inhibit the production of both procollagen I and III by different signaling mechanisms.

However, the downstream signaling pathways that mediate the inhibitory effects of caffeine remain obscure. Therefore, in this study, we also treated HSC-T6 cells with the same scheme described above. Likewise, we found that caffeine significantly inhibited acetaldehyde-stimulated phosphorylation of SRC, ERK1/2, and P38 MAPK. Moreover, the addition of inhibitors of PKA, SRC, and ERK1/2 to the caffeine-treated cells further decreased the level of phosphorylated ERK1/2, and inhibitors of PKA and SRC also significantly down-regulated the phosphorylation of SRC, whereas inhibitor of ERK1/2 did not affect phosphorylation of SRC. In contrast, the simultaneous treatment with caffeine and SB202190 did not interfere with any of these signaling events. These findings confirmed that the sequence of the cAMP-dependent signaling mediated by A2AR is cAMP-PKA-SRC-ERK1/2, which is also most consistent with previous studies in other cells [51]–[53].

Furthermore, our data indicated that P38 MAPK phosphorylation contributed to up-regulation of collagen production and P38 MAPK phosphorylation was reduced more effectively by caffeine plus SB202190 treatment compared with caffeine given alone, but the addition of inhibitors of PKA, SRC, and ERK1/2 to caffeine did not affect caffeine’s inhibitory effect on the phosphorylation of P38 MAPK. Taken together, these findings parallelled the effects of caffeine on procollagen I and III production in HSC-T6 cells, and strongly supported the concept that both cAMP/PKA/SRC/ERK1/2 and P38 MAPK signals contribute to the expression of collagen stimulated by acetaldehyde in HSC. These findings further demonstrated that the inhibitory effects of caffeine on procollagen I are likely to be mediated by PKA-SRC-ERK1/2 pathways downstream of Gs-AC-cAMP, whereas A2AR-P38 MAPK dependent pathway may be involved in mediating the effects of caffeine on the synthesis of procollagen III.

In summary, in this study we used acetaldehyde-induced HSC-T6 cells culture as the in vitro alcoholic liver fibrosis model to investigate the signaling mechanism by which caffeine affect the activation of HSC. The novel finding of this study is that caffeine could suppress acetaldehyde-induced HSC-T6 cells activation by distinct A2AR mediated signal pathway: via inhibition of cAMP-PKA-SRC-ERK1/2 for procollagen type I and via P38 MAPK for procollagen type III. This study provided a new insight into the role of A2AR in the pathogenesis of alcohol-induced liver fibrosis and will help to develop a rationale for the use of A2AR antagonist-based therapy.
